# Achieving Hemostasis Posttranscatheter Aortic Valve Replacement in a Patient with Aortobifemoral Bypass Graft Using Perclose Device: A Novel Technique

**DOI:** 10.1155/2021/4253570

**Published:** 2021-10-26

**Authors:** Ebubechukwu Ezeh, Mohammad Amro, Esiemoghie Akhigbe, Mackenzie Hamilton, Mehrette Maru

**Affiliations:** ^1^Internal Medicine Department, Marshall University, Huntington WV, USA; ^2^Internal Medicine Residency, The Palestinian Ministry of Health and Higher Education, State of Palestine; ^3^Department of Cardiology, St. Mary's Medical Center, Huntington WV, USA

## Abstract

The presence of aortobifemoral bypass graft can complicate vascular access during percutaneous intervention. Choosing an access route for transcatheter aortic valve replacement (TAVR) in this patient population can be challenging. Access options are further limited by the presence of coexisting vascular comorbidities such as extensive peripheral artery diseases in these patients. Adequate preoperative planning to determine the suitability of different access sites is, therefore, very crucial. Our case report shows that the use of Perclose can be a viable option for achieving hemostasis after a direct puncture of an aortobifemoral bypass graft during transfemoral TAVR.

## 1. Introduction

Transcatheter aortic valve replacement (TAVR) has become a common treatment option for patients with severe symptomatic aortic valve stenosis, and a transfemoral (TF) arterial approach is the most common approach [1]. Thus, whenever feasible, the TF should be employed for TAVR because it is more effective than alternative approaches, including transapical, transsubclavian, and direct aortic procedures [2]. Distorted anatomy of the femoral arteries as seen in patients with aortobifemoral bypass graft can, however, make TF access challenging. In this, we present a case where we used successfully used Perclose to close an aortobifemoral graft bypass puncture site during percutaneous transfemoral TAVR.

## 2. Case Presentation

Patient is an 81-year-old male with a history of nonrheumatic aortic stenosis who was admitted for a planned TAVR after complaining of a 6-month history of progressive dyspnea that had markedly impacted his quality of life. He denied palpitation, chest pain, or syncope. Notably, he had past medical history of peripheral artery disease status postaortobifemoral bypass grafting, abdominal aortic aneurysm repair, and lung cancer status postradiotherapy. He also had a history of coronary artery disease status postright coronary artery (RCA) stent. Coronary angiography performed one month prior to admission showed patent RCA stent and stable left coronary disease.

**Figure 1:**
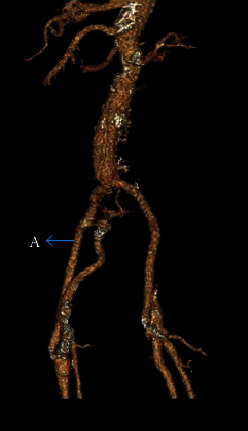
Showing graft (A) and stenosed, native femoral artery.

Upon admission, his vital signs were blood pressure 145/49 millimeter of mercury (mmHg), pulse 68 beats/minute, and oxygen saturation of 96%. Cardiac auscultation revealed a systolic, grade 4 murmur maximally heard at the second right intercostal space and that radiated towards the carotid. Transthoracic echocardiogram confirmed severe aortic stenosis (maximal velocity > 4.0 m/s, and mean gradient > 40 mmHg and aortic valve area 0.8 squared centimeter). Ejection fraction (EF) was 60% with mild left atrial enlargement. His Society of Thoracic Surgeons Predicted Risk of Mortality (STS-PROM) score was calculated to be 19%; he was, therefore, admitted for transcatheter aortic valve replacement (TAVR). Pre-TAVR work up with computed tomography angiogram (CTA) showed subclavian artery stenosis. CTA abdomen and pelvis showed widely patent graft aortobifemoral graft and widely patent femoral bifurcation bilaterally with moderate atheromatous calcification. These are shown in Figure 1.

Considering his comorbidities, the decision was made to utilize the TF approach in accessing the graft using adequate cutaneous anesthesia. TAVR was performed, and Perclose was successfully used to secure the puncture site with no complication. Patient's symptom improved afterwards. He had an uneventful recovery and was discharged.

## 3. Discussion

There have been few cases of TAVR in patients with previous aortobifemoral bypass grafting. Two previous case reports demonstrated the importance of thorough pre-TAVR planning including angiography to identify optimal access [3]. This is especially true in patients with previous aortobifemoral bypass grafting like our patient. Defining the most optimal strategy—as well as bail-out options—for access and closure of the large-bore arteriotomy is an absolute necessity in cases of previous aortobifemoral bypass grafts [3]. Also, peripheral artery disease, which is a major comorbidity in patients referred for TAVR, provides challenge in completing the TF-TAVR procedure in patients with comorbidities associated with femoral access routes such as iliofemoral arteries with severe narrowing, or an artificial bypass graft [4]. In these situations, other access routes like subclavian access are more typically utilized [5]. Our patient had stenosed subclavian artery; thus, we elected to use the TF route despite his history of aortobifemoral bypass graft.

**Figure 2:**
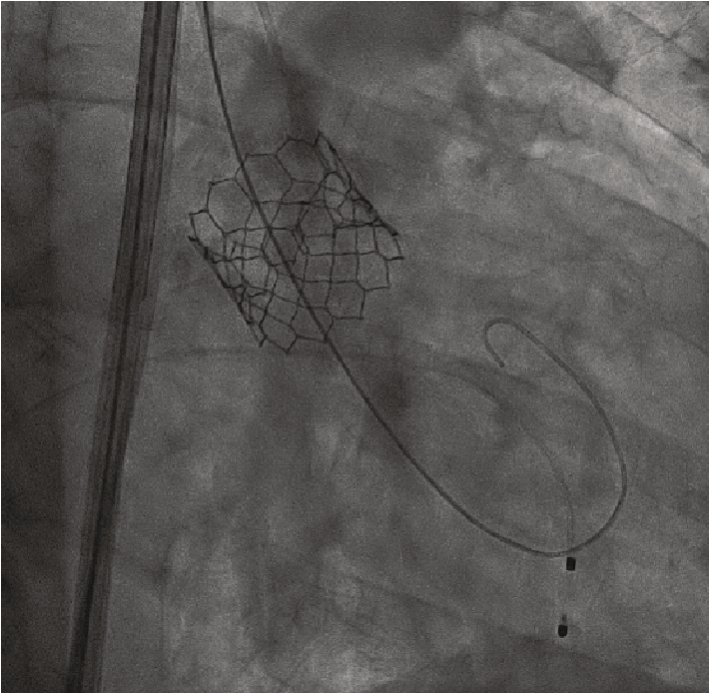
Showing deployed Sapien 3 valve.

**Figure 3:**
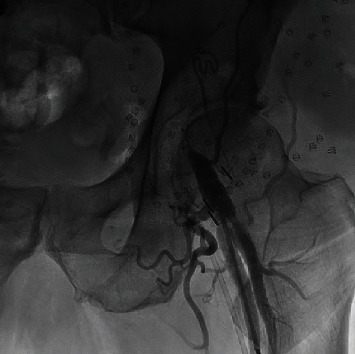
Angiogram showing graft puncture site without complications.

During the procedure, the left common femoral artery was accessed with a needle under ultrasound guidance. A guidewire was introduced followed by a 6-French sheath using a modified Seldinger technique. A transvenous pacing wire was introduced into the right ventricle through the common femoral vein; it was interrogated and was confirmed to be functioning well. Using a guidewire, an angle pigtail catheter was introduced into the ascending aorta through the left common femoral artery. At the root of the aorta, an angiogram was performed to obtain the optimal angle for valve deployment. At this time, the right common femoral artery was accessed with a needle under ultrasound guidance. With a modified Seldinger technique, a guidewire followed by a 6-French sheath was introduced. Then, two Perclose devices, to be deployed at the end of the procedure, were placed at the access sites with the help of a guidewire. After obtaining adequate systemic heparinization, an 8-French sheath followed by a stiff Amplatz guidewire was introduced into the arch of the aorta. Dilators were used after which a 14-French Edwards Sapien sheath was introduced into the thoracic aorta. Then, with the help of a catheter, the guidewire was passed across the aortic valve. After that, a pigtail catheter was introduced into the left ventricle. Subsequently, a 29-millimeter Sapien ultra 3 bioprosthetic valve, which was mounted on delivery system was then introduced with the help of Amplatz guidewire across the aortic valve (Figure 2). The bioprosthetic valve was deployed under fluoroscopy, under rapid pacing when the blood pressure was low. TTE was obtained and confirmed proper positioning of valve with no paravalvular leak. Then, the Perclose devices were completely deployed, and heparin was reversed with protamine. Sterile dressing was applied after the left common artery was closed with angio-seal. Angiogram showed no complication (Figure 3). Immediate postprocedure period was unremarkable.

## 4. Conclusion

Closure of distorted anatomies such as aortobifemoral graft can present a great challenge during TAVR. This case report demonstrates that TF-TAVR with Perclose closure of the femoral access in a patient with vascular endograft is feasible approach—after careful preprocedural imaging, assessment, and planning. As a result, the transfemoral TAVR approach can reasonably be expanded to patients with previous aortobifemoral bypass graft surgery who have contraindications to general anesthesia and/or alternative access routes.
